# The Macklin Effect in COVID-19

**DOI:** 10.7759/cureus.16949

**Published:** 2021-08-06

**Authors:** Noreena Iqbal, Ayesha Malik, Manahil Chaudhry

**Affiliations:** 1 Internal Medicine, Milton Keynes University Trust Hospital, Milton Keynes, GBR; 2 Internal Medicine, Hameed Latif Hospital, Lahore, PAK

**Keywords:** pneumomediastinum, covid-19, macklin effect, pneumothorax, covid complications

## Abstract

From the mere outlook of the ongoing pandemic, coronavirus (severe acute respiratory syndrome coronavirus 2 or SARS-CoV-2) seems to target mainly the respiratory system, but more evolving evidence has advocated its multi-organ involvement. While various complications have been reported in coronavirus disease 2019 (COVID-19) patients, spontaneous pneumomediastinum (SP) remains an uncommon complication.

## Introduction

Coronavirus disease 2019 (COVID-19) infection is largely a self-limiting disease, however, a significant subset of patients; especially those with comorbidities, tend to experience a rather complicated course, even fatality. Some of the well-known complications that can lead to this are the development of acute lung injury, shock, secondary infection, pulmonary embolism or acute kidney injury [1]. 

Due to unreliable sensitivity of reverse transcriptase polymerase chain reaction (RT-PCR), chest X-ray (CXR) and high-resolution computed tomography (HRCT) chest are often used to support a diagnosis and gauge severity in COVID-19 infection [2]. HRCT can show parenchymal changes ranging from ground-glass opacities to widespread consolidation [3]. Seldom have some extra-parenchymal findings such as pneumomediastinum been reported. The authors here present a case of COVID-19 infection complicated by spontaneous pneumomediastinum (SP) with an aim to shed light on this rare complication and explain its pathophysiology by the help of a plausible theory, the Macklin effect.

## Case presentation

**Figure 1:**
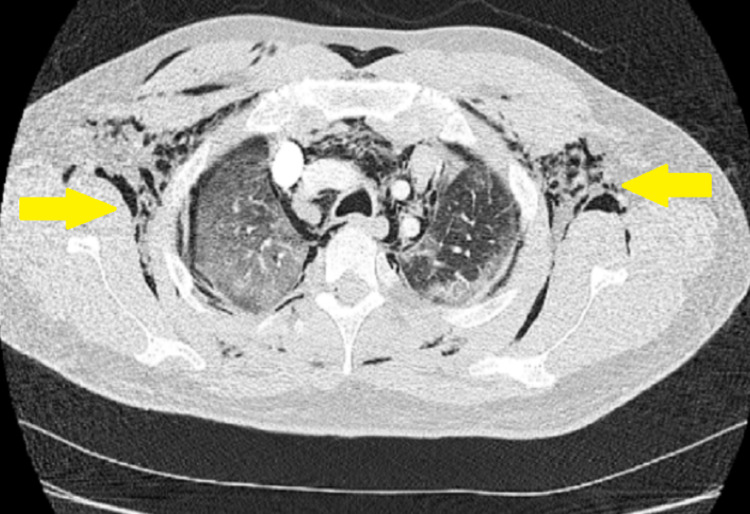
Chest CT axial section of pulmonary parenchymal window showing subcutaneous emphysema (yellow arrows) and extensive ground-glass opacities in the lung parenchyma.

**Figure 2:**
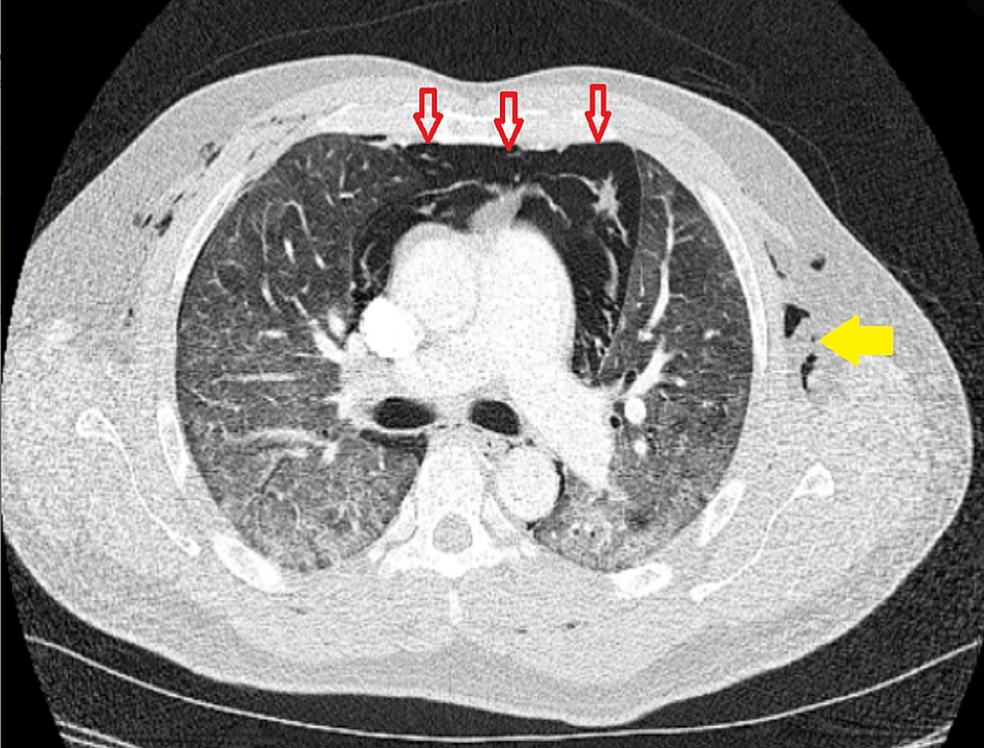
Chest CT axial section of pulmonary parenchymal window showing extensive ground glass opacities in both lung fields, along with subcutaneous emphysema (yellow arrow), and pneumomediastinum (red arrows).

A previously healthy 42-year-old Asian male presented to the emergency department with a 10 days history of fatigue, dry cough, and two days history of high-grade fever along with shortness of breath. On presentation, his oxygen saturation was 60% on room air, heart rate was 118 per minute, blood pressure of 130/80 mmHg, and respiratory rate of 28 per minute. Auscultation of the chest revealed bilateral basal fine crepitation, central trachea, and crepitus on palpation extending from each clavicle to the mandible bilaterally and extending down to the left arm. His jugular venous pulse was not raised and there was no pedal or sacral edema. The rest of the systemic examination was unremarkable. He denied any chest pain, recent long-distance travel, leg swelling, or rash. There was no indication of bowel or esophageal rupture. He denied the use of any recreational drugs and was a non-smoker. On admission, he was started on oxygen supplementation at 15 liters/min via a non-rebreather mask along with, dalteparin (prophylaxis), and broad-spectrum antibiotics. His blood work revealed normal full blood count, urea, and electrolytes. However, his C-reactive protein was 624 mg/L (0-6 mg/L), d-dimers 2041 ng/ml (0-230 ng/ml), and his RT-PCR for SARS-CoV-2 came back positive. In view of the high d-dimer value, we proceeded with a contrast-enhanced computed tomography pulmonary angiogram (CTPA) to rule out pulmonary embolism. The study was negative for pulmonary embolism, but it revealed more than just parenchymal changes consistent with COVID pneumonia i.e. bilateral consolidation at lung bases, extensive pneumomediastinum, surgical emphysema throughout the chest wall, and bilateral small pneumothoraces (Figures 1-2).

A multidisciplinary team’s opinion was to manage conservatively and in case of desaturation, humidified high flow oxygen was planned to be delivered in the intensive therapy unit. He responded well to conservative treatment with a gradual reduction in oxygen requirement. He was eventually weaned off of supplemental oxygen, never having to be mechanically ventilated, and was discharged on day 12. He was advised follow-up after six weeks.

## Discussion

The exact mechanism of SP in non-ventilated patients remains unknown. However, the Macklin effect has been proposed as a possible etiology [3] owing to the SARS-CoV-2 propensity to damage type 2 pneumocytes [4]. The Macklin effect starts with alveolar rupture secondary to direct alveolar injury, leading to air leaking and dissection along the bronchovascular sheaths and eventually spreading of air within the mediastinum [5]. This leaking of air can be aggravated by intense coughing leading to a sudden increase in distal airway pressure and ultimately alveolar rupture [6]. The air-leak can also spread to the fascial planes of the neck and chest leading to subcutaneous emphysema, which was also a manifestation in our patient. The most common symptom of pneumomediastinum is acute retrosternal chest pain [4] and should warrant early alert to rule out this dreaded complication in patients with suspected or confirmed COVID-19. However, our patient did not complain of retrosternal chest pain. The treatment for SP is usually symptomatic and conservative. Oxygen therapy could possibly lead to faster recovery [7]. 

Loffi and colleagues consecutively studied 102 patients and found the incidence of SP to be 6%, and reported a mortality of one in six patients [3]. They also noted that there was no statistical difference in clinical severity in those with and without pneumomediastinum. Mimouni et al. and Mohan et al. [4,8] described cases of a 65-year-old male and a 49-year-old male, respectively, who deteriorated while being in-patient, and later a confirmed diagnosis of spontaneous pneumomediastinum was made with further imaging. Lastly, Volpi and colleagues described three severe cases of COVID-19 infection who required mechanical ventilation but later made full recovery and were discharged [9]. Unlike these cases, our patient improved on conservative, high-flow oxygen treatment. 

Although isolated pneumomediastinum might be a self-limiting condition, upon literature review we noted that in patients who had concurrent pneumopericardium, the clinical course was more severe and outcomes, less favorable [10-12]. This could likely be due to a more severe hemodynamic risk associated with the presence of pneumopericardium. Juárez-Lloclla and colleagues studied 12 COVID-19 patients that developed SP and/or pneumopericardium and compared their clinical presentation, complications, and outcomes during hospitalisation. A higher mortality rate was reported in their study (six out of eleven patients who had pneumopericardium along with pneumomediastinum) [13]. Pneumopericardium can be thought of as an extended complication of the Macklin effect. 

## Conclusions

Extra-parenchymal pulmonary findings are a rare complication seen in COVID-19 patients. No set guidelines have been devised yet for the treatment of SP and pneumopericardium in COVID-19. However, the management is largely conservative with increased mortality reported in patients with concurrent pneumopericardium. Any suddenly deteriorating patient with increased oxygen requirements should raise suspicion of an underlying SP, prompting early chest imaging.
